# The complete mitochondrial genome and phylogenetic analysis of *Hiatella* sp. J (Mollusca: Hiatellidae)

**DOI:** 10.1080/23802359.2020.1763217

**Published:** 2020-05-13

**Authors:** Hao Wang, Mingxuan Teng, Jing Liu, Haoyuan Pan, Qifan Zeng, Shi Wang, Zhenmin Bao

**Affiliations:** a MOE Key Laboratory of Marine Genetics and Breeding, College of Marine Life Sciences, Ocean University of China, Qingdao, China; b Laboratory for Marine Fisheries Science and Food Production Processes, Qingdao National Laboratory for Marine Science and Technology, Qingdao, China; c Laboratory for Marine Biology and Biotechnology, Qingdao National Laboratory for Marine Science and Technology, Qingdao, China

**Keywords:** *Hiatella*, mitogenome, bivalve, phylogeny

## Abstract

The genus *Hiatella* is one of most abundant and widespread marine bivalves. To date, its intra-generic phylogeny remains disputed and mitogenome information is therefore much needed. Here, we first report the complete circular mitogenome of *Hiatella* sp. J that is distributed in the coast of Asia Pacific. The total length of this mitochondrial genome is 21,233 base pairs. It consists of 13 protein-coding genes, 22 transfer RNAs, 2 ribosomal RNAs, and a major noncoding region (MNR). Phylogenetic analysis of COI from 25 species (Hiatellidae) revealed that the *Hiatella* sp. J was closely related to Asian *Hiatella* in the family Hiatellidae. This *Hiatella* mitogenome provides new molecular data for the further taxonomic and phylogenetic studies of the genus *Hiatella* of marine bivalves.

The marine bivalve genus *Hiatella* is extremely abundant with high diversity in morphology and great plasticity in the mode of life history. As their shell shapes and modes of life history are weakly correlated with the suggested taxonomical identity, there is still no generally accepted intra-generic phylogeny exists at the moment (Hunter 1949; Coan et al. 2000). The *Hiatella arctica* has long been considered as a cosmopolitan species that comprises multiple lineages (Lubinsky 1980). Previous study of Laakkonen et al. (2015) revealed that at least 13 of the recognized lineages represent distinct species. According to the phylogenetic evidence of mitochondrial COI, nuclear ANT and 28S rRNA genes, the *Hiatella* sp. J lineage in the east coast of Asian Pacific is a new *Hiatella* species (NCBI: txid1638755) (Laakkonen et al. 2015). To better understand the phylogenetic relationships of Hiatellidae, here we present the complete mitochondrial genome of *Hiatella sp.* J.

An individual sample of *Hiatella* sp. J was collected at the Laizhou Bay in Shandong province and deposited in our laboratory collection (voucher specimen number: OUC-MGB-2018-HO-01). The muscle tissue samples were collected, washed with 3.5× concentrated PBS, and preserved in liquid nitrogen. Total genomic DNA was extracted using a phenol-chloroform extraction protocol (Russell and Sambrook 2001). DNA sequencing was carried out using genomic DNA by an Illumina Xten platform. *NOVOPlasty* (Dierckxsens et al. 2017) and *MITOS* (Bernt et al. 2013) were utilized for mitogenome assembly and annotation, respectively. Maximum-likelihood (ML) was conducted on the partitioned matrices with *FastTree* (Price et al. 2009).

The complete circular mitogenome was 21,233 bp in length with 35.9% of GC content. It contained 13 protein-coding genes, 24 transfer RNAs, and 2 ribosomal RNAs. The mitogenomic sequence was submitted to GenBank with the accession number *MN072638*. Phylogenetic interference was performed with the COI genes of 22 *Hiatella* specimens (Laakkonen et al. 2015), outgrouped by two *Panopea* species. The ML phylogenetic tree depicted that our sample clustered with the *Hiatella* sp. J (Figure 1), and had a close relationship with *Hiatella* sp. L that is distributed in Bering strait, Arctic ocean, Alaska, etc. This study provides a valuable mitogenome resource for better understanding of the molecular phylogeny of the genus *Hiatella* of marine bivalves.

**Figure 1.**
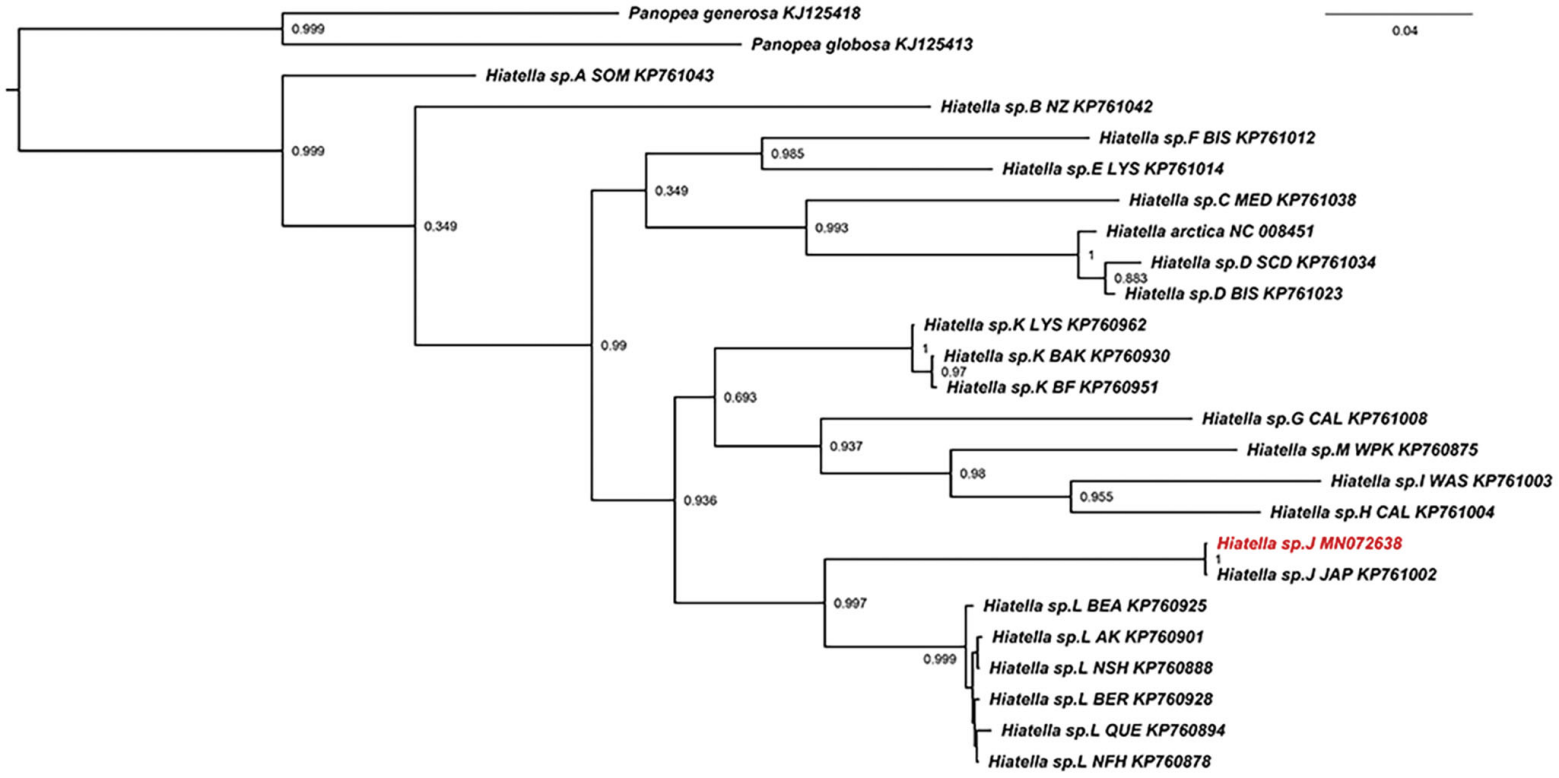
The phylogenetic tree of COI based on ML. The accession number and sample sites for these species are as follows: *Hiatella* sp. L NFH (KP760878); *Hiatella* sp. L NSH (KP760888); *Hiatella* sp. L QUE (KP760894); *Hiatella* sp. L AK (KP760901); *Hiatella* sp. L BEA (KP760925); *Hiatella* sp. L BER (KP760928); *Hiatella* sp. J JAP (KP761002); *Hiatella* sp. M WPK (KP760875); *Hiatella* sp. K BAK (KP760930); *Hiatella* sp. K BF (KP760951); *Hiatella* sp. K LYS (KP760962); *Hiatella* sp. I WAS (KP761003); *Hiatella* sp. H CAL (KP761004); *Hiatella* sp. G CAL (KP761008); *Hiatella* sp. F BIS (KP761012); *Hiatella* sp. E LYS (KP761014); *Hiatella* sp. D BIS (KP761023); *Hiatella* sp. D SCD (KP761034); *Hiatella* sp. C MED (KP761038); *Hiatella* sp. B NZ (KP761042); *Hiatella* sp. A SOM (KP761043); *Panopea generosa* (KJ125418); *Panopea globosa* (KJ125413); *Hiatella arctica* (NC 008451).

## Data Availability

The data that support the findings of this study are openly available in GeneBank at [https://www.ncbi.nlm.nih.gov/nuccore/MN072638.1/], reference number [Accession number: *MN072638*].
